# Intermittent Episodes of Bright Light Suppress Myopia in the Chicken More than Continuous Bright Light

**DOI:** 10.1371/journal.pone.0110906

**Published:** 2014-10-31

**Authors:** Weizhong Lan, Marita Feldkaemper, Frank Schaeffel

**Affiliations:** 1 Section of Neurobiology of the Eye, Center for Ophthalmology, University of Tuebingen, Tuebingen, Germany; 2 Zhongshan Ophthalmic Center, State Key Laboratory of Ophthalmology, Sun Yat-sen University, Guangzhou, China; 3 Graduate School of Cellular & Molecular Neuroscience, University of Tuebingen, Tuebingen, Germany; Lund University, Sweden

## Abstract

**Purpose:**

Bright light has been shown a powerful inhibitor of myopia development in animal models. We studied which temporal patterns of bright light are the most potent in suppressing deprivation myopia in chickens.

**Methods:**

Eight-day-old chickens wore diffusers over one eye to induce deprivation myopia. A reference group (n = 8) was kept under office-like illuminance (500 lux) at a 10∶14 light∶dark cycle. Episodes of bright light (15 000 lux) were super-imposed on this background as follows. Paradigm I: exposure to constant bright light for either 1 hour (n = 5), 2 hours (n = 5), 5 hours (n = 4) or 10 hours (n = 4). Paradigm II: exposure to repeated cycles of bright light with 50% duty cycle and either 60 minutes (n = 7), 30 minutes (n = 8), 15 minutes (n = 6), 7 minutes (n = 7) or 1 minute (n = 7) periods, provided for 10 hours. Refraction and axial length were measured prior to and immediately after the 5-day experiment. Relative changes were analyzed by paired t-tests, and differences among groups were tested by one-way ANOVA.

**Results:**

Compared with the reference group, exposure to continuous bright light for 1 or 2 hours every day had no significant protective effect against deprivation myopia. Inhibition of myopia became significant after 5 hours of bright light exposure but extending the duration to 10 hours did not offer an additional benefit. In comparison, repeated cycles of 1∶1 or 7∶7 minutes of bright light enhanced the protective effect against myopia and could fully suppress its development.

**Conclusions:**

The protective effect of bright light depends on the exposure duration and, to the intermittent form, the frequency cycle. Compared to the saturation effect of continuous bright light, low frequency cycles of bright light (1∶1 min) provided the strongest inhibition effect. However, our quantitative results probably might not be directly translated into humans, but rather need further amendments in clinical studies.

## Introduction

Nearsightedness (myopia) arises from a mismatch between the focal power of the optical components (cornea and crystalline lens) and the axial length. It is the most commonly found disorder in the development of the juvenile eye and steadily rises in prevalence, currently affecting 30–50% of young adults in Europe [1], [2] and around 80% in Asia [3], [4]. Recent studies have shown that outdoor exposure seems to be a promising approach to reduce the development of myopia – children who spend more time outdoors appear to be less likely to become myopic [5]–[10].

A number of possible factors can be suggested for the protective effect of outdoor exposure, such as light intensity, physical activity, viewing distance, variations in accommodative requirement, which have been systematically discussed by a recent review [11]. Rose et al. were the first to suggest that light intensity might be an important factor [8], [12] and this assumption has gained accumulating experimental evidence in animals. Specifically, with the urbanization of modern world, humans tend to spend more time indoors with illuminances typically ranging from 100 lux to 500 lux. Compared with the outdoor illuminance (as high as 150 000 lux on a sunny summer day), the indoor illuminance is very much lower. Cohen et al., observed that chickens raised at low light (50 lux) for extended periods (90 days) developed significant myopia (Mean: −2.41D), as compared to those reared under standard (500 lux, Mean: +0.03D) or high light level (10 000 lux, Mean: +1.1D). [12] Furthermore, exposure to artificial bright light (15 000 lux to 25 000 lux) has been shown to suppress deprivation myopia that is induced by covering the eye with frosted diffusers in chickens [13], tree shrews [14] and monkeys [15]. Bright light also slows down the development of myopia induced by wearing negative lenses in chickens [16] and tree shrews [14], [17], although no significant effects were observed in monkeys in the only study done so far[18]. Overall, there is now convincing evidence to support the speculation proposed by Norton and Siegwart that “ambient illuminance levels produce a continuum of effects on normal refractive development and the response to myopiagenic stimuli such that low light levels favor myopia development and elevated levels are protective” [17].

There are currently three trials on prevention of myopia with outdoor exposure [19]–[21]. All three trials reported statistically significant reduction in the incidence rate and the progression rate of myopia with increasing time outdoors. If the protective effect of the outdoor exposure is attributable to light intensity, given the fact that bright light was found to inhibit myopia in several different species, it is likely that a simple myopia therapy in children might be to increase ambient illuminance in classrooms. However, there is still much to be learned about the exposure parameters that have the greatest inhibitory effect on the development of myopia [15]. In particular, the dose-response function has not been explored and nor have the optimum temporal patterns for exposure to bright light been determined. In the current study, we have tackled some of these questions, using the chicken model of myopia.

## Materials and Methods

### Animals

One-day-old male white leghorn chickens were obtained from a local hatchery in Kirchberg, Germany. They were raised in a temperature-controlled room under 500 lux ambient illuminance with a 10/14 hour light/dark cycle (light on at 8AM and off at 6PM). Chickens had free access to food and water. All experiments were conducted at the University of Tuebingen. This study was carried out in strict accordance with the ARVO Statement and the guide of the regional council of Tuebingen for the care and use of laboratory animals. The protocol was approved by the Regional Council of Tuebingen (Reference number: AK 3/12). All efforts were made to minimize suffering during the study and chickens were sacrificed via ether after the experiments.

### Experimental Paradigms

From the day 8 post-hatching, frosted diffusers were placed over chickens' right eyes to induce monocular deprivation myopia, a common model for human myopia [22]–[26]. Chickens were then randomly assigned to one of the following two experimental paradigms. In both paradigms, an illuminance of 500 lux served as constant background.

#### Paradigm I

Chickens were exposed to constant bright light (approximately 15 000 lux) for either 5 hours (from 10AM to 3PM, n = 4) or 10 hours (the entire light phase, n = 4) per day. To determine the minimum duration with a significant effect on myopia, exposure durations of 1 hour (from 12∶30 AM to 1∶30 PM, n = 5) or 2 hours (from 12AM to 2 PM, n = 5) were also tested.

#### Paradigm II

Chickens were exposed to intermittent bright light (approximately 15 000 lux) with a 50% duty cycle and either 60 minutes (n = 7), 30 minutes (n = 8), 15 minutes (n = 6), 7 minutes (n = 7) or 1 minute (n = 7) cycle length over a period for 10 hours. Thus, the total daily duration of exposure to bright light was 5 hours in all cases.

In addition, two control groups with four animals in each were kept under background illuminance without further interventions, except for wearing frosted diffusers over their right eyes. Two different batches were used to evaluate inter-batch variability but it turned out to be negligible (see Results).

All treatments were continued for 5 consecutive days. Details about the spectral energy distribution of the two light sources have been described previously [13]. The emission spectra of the lamps were similar to the spectrum of the sun over the visible range of wavelengths. Air conditioners were applied to match the environmental temperature in the groups of the two paradigms (range 25–27°C).

### Measurement of Ocular Parameters

Ocular parameters were measured both prior to and immediately after the 5-day treatment period. Refractions were determined by automated infrared photoretinoscopy without cycloplegia [27], and ocular biometry was performed by A-scan ultrasonography with a probe of 10 MHz [28].

### Statistics

Data are presented as the mean ± one standard error of the mean (SEM). Relative changes between deprived eyes and non-deprived eyes within a group were compared with paired t-tests. Comparisons among groups were assessed by one-way ANOVA, with post-hoc protected Fisher Least Significant Difference (LSD) pairwise multiple comparisons. If necessary, the absolute changes of ocular parameters over time or between two individual groups were tested with paired t-tests and unpaired t-tests respectively. All analyses were performed with commercially available software (SPSS 16.0; SPSS, Chicago, IL). Tests of significance were two-tailed, and the level of significance was set at 0.05.

## Results

### Refractive errors

As expected, after 5 days of diffuser wear, the covered eyes developed significant myopia in both reference groups (group 1: −10.84±1.09D, t = −9.985, P = 0.002 and group 2: −10.75±1.81D, t = −5.927, P = 0.010). There was no significant difference between the groups (t = −0.043, P = 0.967). Therefore, the data of both groups were pooled to generate a reliable reference group against which the effects of bright light exposure could be tested.

**Figure 1:**
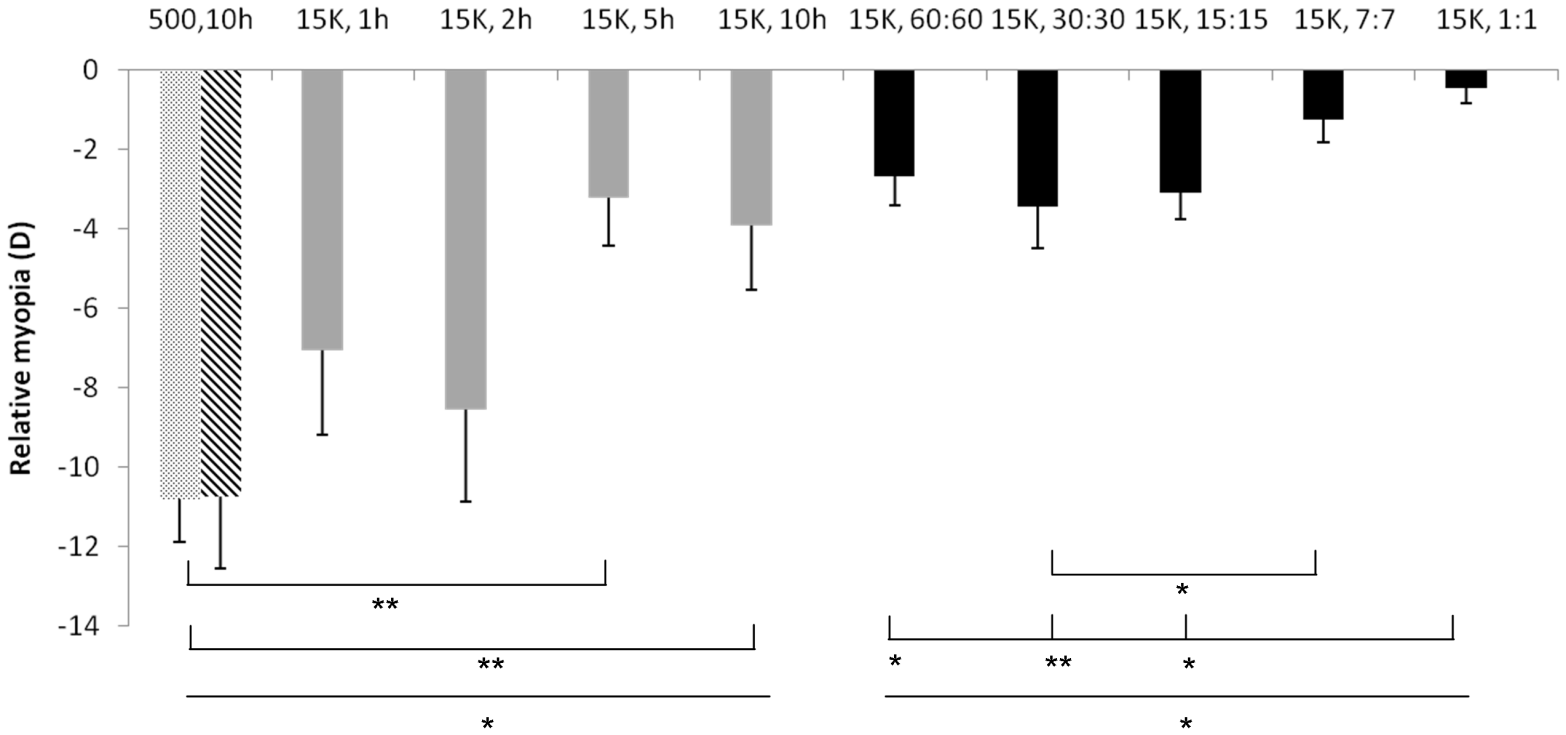
Myopia induced by diffusers over one eye when chickens were kept under constant bright light of 15 000 lux for 1, 2, 5, or 10 hours (“15k, time in hours”; filled gray bars) or under cycles of bright light, changing from 500 to 15 000 lux at different frequencies (“15k, half cycle duration; black bars). Patterned gray bars show the amount of myopia that developed in two batches of chickens wearing monocular diffusers under regular laboratory illumination of 500 lux (“500,10 h”). Because there was no difference between both groups, their data were pooled and provided the reference for the bright light treatment groups. An inhibitory effect of constant bright light was observed only when the exposure lasted for 5 hours or more (P = 0.004 and P = 0.007 for 5 and 10 hours, respectively). No additional benefit was observed when the bright light exposure was extended to 10 hours, compared with those exposed to 5 hours (P = 0.796). When bright light was provided as a temporal square wave function, its protective effect against myopia was enhanced. Chickens kept under 7∶7 or 1∶1 minute cycles developed the least myopia, compared with other cycles (P = 0.033 for differences among groups reared under cycles of bright light; Post-hoc pairwise comparison: 7∶7 minutes vs 30∶30 minutes, P = 0.038; 1∶1 minutes vs 60∶60, 30∶30, 15∶15 minutes: P = 0.041, 0.006, 0.022, respectively). *<0.05, **<0.01.

Varying durations of continuous bright light (paradigm 1) inhibited deprivation myopia to different extents (F = 3.817, P = 0.017; Figure 1). Post-hoc analysis revealed that exposure for 1 or 2 hours did not provide significant protection against myopia development (P = 0.099 and P = 0.309, respectively). Significant inhibition of myopia was observed only when exposure duration was extended to 5 hours or more (P = 0.004 and P = 0.007 for 5 and 10 hours, respectively). Nevertheless, there was no significant difference between groups reared with 5 or 10 hours of bright light (P = 0.796).

In paradigm 2, bright light was applied as a temporal square wave function (changing repeatedly between 500 and 15000 lux). It was found that the protective effect of bright light was enhanced when the cycles were in the range of minutes (Figure 1). Still, no difference was found when bright light was applied continuously for 5 hours or in episodes of 60∶60 minutes for 10 hours (−3.23±1.19D vs −2.70±0.73D, t = −0.404, P = 0.696). However, significant difference was detected among the groups exposed to repeated cycles of bright light (Paradigm 2, F = 3.023, P = 0.033). Post-hoc tests revealed that myopia inhibition was maximal when chickens were kept at 7∶7 minutes and 1∶1 minutes cycles (7∶7 minutes vs 30∶30 minutes: P = 0.038; 1∶1 minutes vs 60∶60, 30∶30, 15∶15 minutes: P = 0.041, 0.006, 0.022, respectively). For 1∶1 minute cycles, the degree of induced myopia was significantly lower than that after 5 hours of continuous bright light (−3.23±1.19D vs −0.47±0.38D; t = −2.749, P = 0.023).

Interocular differences in the refractive errors in chicks kept under different light cycles are shown in Table 1. Under 60∶60 to 15∶15 minute cycles, significant myopia developed in the deprived eyes (all P<0.05). However, there was no longer a significant interocular difference in the refractive errors when the animals were under 7∶7 and 1∶1 minute cycles (P = 0.066 and 0.256, respectively).

**Table 1:** Interocular differences in myopia and the depth of the vitreous chamber of the eyes (VCD) with monocular diffusers under different temporal cycles of bright light.

Group	N	Relative myopia		Relative VCD elongation	
		Mean±SEM	P	Mean±SEM	P
60∶60	7	−2.70±0.73D	0.010*	0.24±0.07	0.012*
30∶30	8	−3.45±1.04D	0.013*	0.34±0.09	0.007*
15∶15	6	−3.09±0.66D	0.005*	0.28±0.08	0.017*
7∶7	7	−1.27±0.56D	0.066	0.20±0.07	0.032*
1∶1	7	−0.47±0.38D	0.256	0.05±0.06	0.428

*significant myopic shifts in deprived eyes compared to non-deprived fellow eyes.

### Ocular biometry

**Figure 2:**
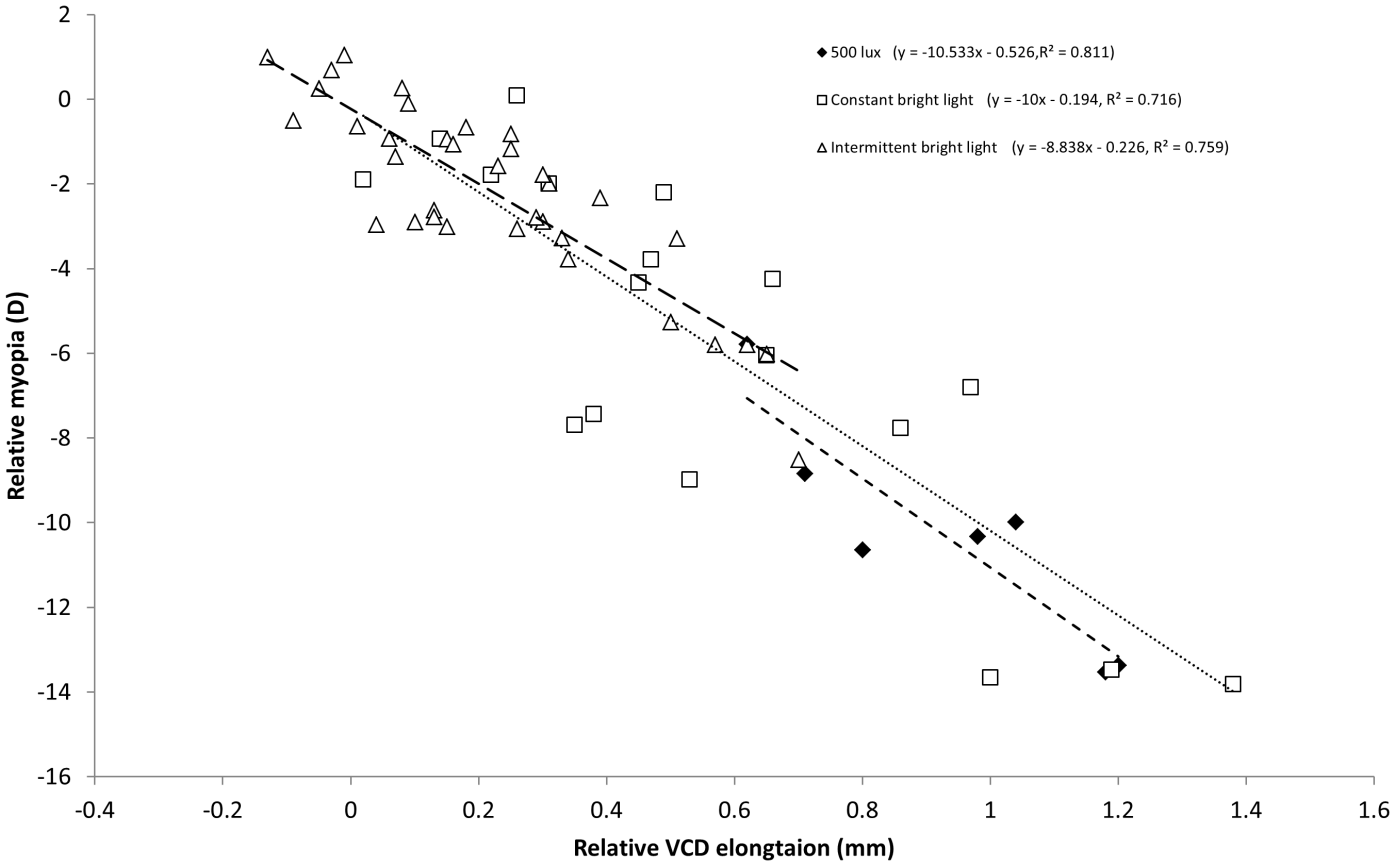
Correlation between vitreous chamber depth and the amount of myopia in chickens under different light regimens. Equations for the linear regression, and R^2^ values are provided for each light regimen. Long dash line represents the data for intermittent bright light, dotted line for constant bright light and short dash line for standard illuminance, respectively. Note that one diopter of myopia was equivalent to about 0.1 mm of axial elongation across groups (data from one single animal were excluded from the plot because of apparent measurement error, data: −13.9D vs 0.25 mm).

In the two reference groups, vitreous chamber depth (VCD) increased about linearly with the amount of myopia with about 0.1 mm per diopter of myopia (R^2^ = 0.811, P = 0.005, Figure 2). There was no significant difference between both groups (t = −0.750, P = 0.487). The ratio of vitreous chamber elongation to increase of myopia was similar among the different groups raised in continuous bright light (R^2^ = 0.716, P<0.001) or in intermittent bright light (R^2^ = 0.759, P<0.001).

**Figure 3:**
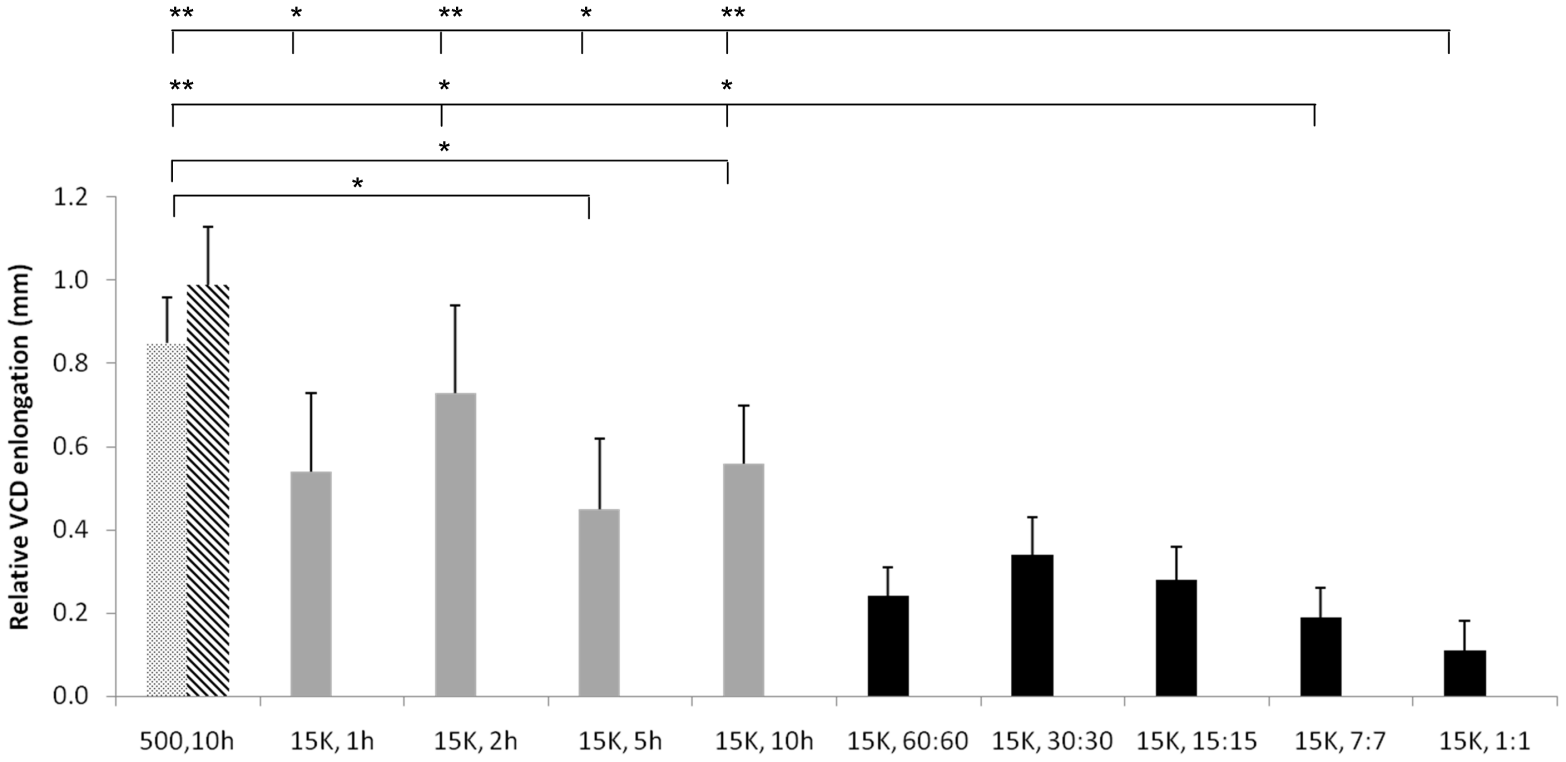
Relative increase in vitreous chamber depth (VCD) in eyes with monocular diffusers (bars grey-scale coded as in Figure 1). Although there was no significant difference among treatment groups for either paradigm (Paradigm I: F = 1.639, P = 0.204 and Paradigm II: F = 2.075, P = 0.109), the increase of VCD in chickens reared under constant bright light for 5 or 10 hours was signifcantly supressed compared with those under standard illuminance (P = 0.029 and 0.037, respectively). In comparison with constant bright light, this effect was further enhanced in chickens exposed to cycles of bright light at a frequency of 7∶7 or 1∶1 minutes (all P<0.05, except for the comparison between the 7∶7 minute cycle and the 5 h constant bright light exposure, P = 0.180 and a borderline significance between the 7∶7 minute cycle and the 1 h constant bright light exposure, P = 0.075).* <0.05, **<0.01. Abbreviations as in Figure 1.

Interestingly, the changes in vitreous chamber depth due to exposure to the different light regimens were often not as significant as the refractive errors (Paradigm I: F = 1.639, P = 0.204; Paradigm II: F = 2.075, P = 0.109, significances refer to the differences among groups in Paradigm I and II, respectively). However, consistent with previous studies[13], [16], if the data from chickens exposed to 5 or 10 hours of constant bright light was compared to those of the reference group separately, statistical significance was detected (0.45±0.20 mm vs 0.93±0.09 mm, t = −2.590, P = 0.029; 0.56±0.13 mm vs 0.93±0.09 mm, t = −2.451, P = 0.037, respectively). More importantly, it is clear that repeated cycles of bright light generated generally shorter vitreous chambers than constant bright light. Exposure to 7∶7 or 1∶1 min bright light cycles inhibited axial eye growth more than constant bright light (all P<0.05, except for the comparison between the 7∶7 minute cycle and the 5 h constant bright light exposure, P = 0.180 and a borderline significance between the 7∶7 minute cycle and the 1 h constant bright light exposure, P = 0.075). Similar to refractive error, vitreous chamber elongation was almost completely suppressed in the diffusertreated eyes (Figure 3; Table 1) exposed to a 1∶1 min bright light cycle.

No significant changes were detected in anterior chamber depth and lens thickness (LT) between the deprived eyes and the non-deprived eyes, regardless of treatment group (all P>0.05). There were also no differences in these two parameters among groups (F = 0.478, P = 0.752 and F = 0.363, P = 0.833 for ACD; F = 0.024, P = 0.999 and F = 0.258, P = 0.903 for LT, respectively).

## Discussion

Our data show that exposure to continuous bright light of 15 000 lux for 1 or 2 hours every day is not sufficient to provide significant protection against deprivation myopia in the chicken model of myopia. Inhibition of myopia was significant after 5 hours of bright light exposure but, extending the duration to 10 hours, did not offer additional benefit. However, repeated cycles of 1∶1 minutes of bright to standard laboratory light (15 000 versus 500 lux) enhanced the protective effect against myopia and could finally suppress its development completely.

### Should children be exposed to continuous bright light for longer periods of time?

In the first paradigm, we found that 5 hours of 15 000 lux inhibited deprivation myopia by approximately 70%, similar to what was found in different animal models in previous studies with an ambient illuminance of 15 000 to 25 000 lux for 5 to 6 hours [13]–[16]. Short term bright light exposure for 1 or 2 hours generated only a trend towards inhibition of deprivation myopia. This result is consistent with a recent study in chickens in which bright light of 10 000 lux was provided for 2 hours a day but no significant effects were found, no matter at which time of the day it was applied [29]. In comparison, human studies show that children appear to be more “sensitive” to bright light exposure, as Jones et al. observed a marked reduction in the risk of myopia when the amount of time outdoors increased from 0–5 hours per week (approximately 1 hour per day) to>14 hours per week (approximately 2 hours per day) [9]. The three outdoor clinical trials also suggest that significant protection from myopia is also achieved with only 1–2 hours of outdoor exposure per day [19]–[21]. We speculate that the discrepancy between human data and animal studies might be due to, other than species differences, several critical differences in the “treatment protocols”, such as differences in the visual environment, in the procedures to induce myopia, in the shape of the dioptric space, and the durations of the intervention, and their relation to the life span of humans and chicks. Finally, sample size could be another essential factor to consider. It is noted that the sample size of these clinical trials is much higher than the number of chickens in the current study (e.g., 1903 kids were enrolled in the trial launched in Guangzhou [21]). However, in a normal animal study, like the current one, ethical limitations usually prevent the usage of larger numbers of experimental animals.

We also found that no additional benefit was achieved when the exposure duration was extended to the entire light phase (10 hours). Therefore, we assume that there might be a plateau for the protective effect from certain level of dose for continuous bright light exposure, at least in the case of 15 000 lux. If this finding was applicable to children, then the treatment effect for this strategy might have an upper limit. Certainly, it is important to know where the optimal treatment exposure duration is located in children, as the prolonged exposure to bright light would increase the energy consumption (in the case of using artificial lighting). Additional benefit in terms of a complete inhibition of deprivation myopia development in chicks was demonstrated when illuminance level was further increased to 40 000 lux (Ashby, unpublished data), perhaps another approach to enhance the suppressive effect of bright light on myopia. Since our eyes were developed in the course of evolution to operate optimally at day light, there is no reason to assume that 15 000 lux indoors are deleterious to our retina. But a longer exposition to bright light outdoors might also increase the risk of potential side effects, such as skin cancer [30] and retinal light damage (in the case of overexposing to sunlight). [31]

### Should children be exposed to cycles of bright light at low frequencies to have a larger effect on myopia?

In the second paradigm, we replaced the continuous bright light regimens with intermittent ones. Interestingly, providing bright light in pulses with low temporal frequency further suppressed the development of deprivation myopia. Inhibition appears to be frequency-dependent. When the temporal frequency reached 0.001 Hz (7∶7 min cycles of bright to standard light), the differences in refractive error between eyes with normal visual experience and eyes with diffusers were no longer significant, indicating that deprivation myopia was completely suppressed. Even though our findings might be applicable to children, compliance must be considered. In particular, exposure to alternating illuminance between 500 lux and 15 000 lux with short cycles may be less comfortable than constant bright light. Future studies should test whether one really needs 15 000 lux provided at low frequency cycles to fully suppress myopia development. If low frequency flicker at lower light intensity (e.g. 2 000 lux) would have a similar effect, feasibility would be greatly improved.

### Possible mechanisms by which intermittent bright light could inhibit deprivation myopia

As reviewed by French et al., [11] two factors are currently discussed that might be important for the suppression of human myopia by bright light. One is that UV exposure is important since it triggers vitamin D production in the skin; the other is that dopamine release from the retina is stimulated by bright light and has an inhibitory effect on axial eye growth. In favor of the first mechanism, Vitamin D was lower in myopes than non-myopes [32], [33]. On the other hand, evidence against this hypothesis is that feeding tree shrews with a sufficient dose of Vitamin D3 supplements [34] or rearing chickens under bright UV light [35] did not prevent experimental myopia. Furthermore, our finding that deprivation myopia was significantly inhibited by light that was free of UV (cut-off at around 400 nm) also weakens this hypothesis.

By contrast, there is more evidence supporting the hypothesis that dopamine release is stimulated and inhibits axial eye growth. In the first place, dopamine release is known to be almost linearly related to the logarithm of the ambient lighting level [12], [36]–[39]. Furthermore, it has been speculated by Norton and Siegwart [17] that as illuminance levels rise, activation of intrinsically photoresponsive retinal ganglion cells (ipRGCs) might provide an additional way to stimulate the dopamine release, given the finding that ipRGCs synapse directly on dopaminergic amacrine cells in the retina [40]. In parallel, it is known since 1989 that the synthesis and release of dopamine is reduced during the development of deprivation myopia [41]–[43]. Dopamine agonists injected into the vitreous can inhibit deprivation myopia in different species, including chickens [44]–[47], rabbits [48] and rhesus monkeys [49]. On the contrary, spiperone, a dopamine antagonist, was found to block the beneficial effects of bright light on deprivation myopia [16]. In summary, the second hypothesis appears more likely that high illuminances stimulate dopamine release from the retina and that dopamine has an inhibitory effect on axial eye growth [11], [17].

Since low frequency bright flicker light inhibited myopia more than continuous bright light in the present study, one could assume that dopamine release is further stimulated. It was found already in 1987 that flickering light with 10 Hz inhibits deprivation myopia in chickens [50]. Several studies found that flickering light can stimulate the release of dopamine from the retina [51]–[53]. Flickering light results in a strong stimulation of both ON and OFF pathways. Retinal ON-pathway neurons, including dopamine-releasing neurons, respond to the onset of light with a pronounced depolarizing transient that decays to a relatively low plateau level. It is possible therefore that flickering stimuli produces repeated ON-transients which might result in a greater overall release of dopamine than a steady light stimulus [9], [54]. However, there is also evidence that steady light causes more dopamine release [37], [55], [56]. Dong and McReynolds [54] speculated that the inconsistency across these studies might be due to the fact that the light responses of retinal neurons often change dramatically with light intensity or the state of adaptation. For example, most of the studies that reported a larger effect of flickering light were done in light-adapted retinas and with bright light pulses, while those that reported a weaker effect of flickering light used dark-adapted retinas and relatively dim stimuli. In the current study, we used illuminance where the chickens were light adapted and one would assume that dopamine release is enhanced by the flickering light. Nevertheless, it should be pointed out that the current “flickering light” was in a much lower frequency band (0.007 Hz) than flickering light in other studies (1–20 Hz). Thus, it is not clear to what extent the findings from flickering light can be applied to the current light treatments. Further work needs to demonstrate that dopamine release is actually enhanced when flicker frequencies are very low.

We did not consider the effects of light on pupil constrictions, which would add transient components to the retinal illumination when a temporal square wave pattern of light was applied. However, even if the pupil constricts by 50% at the onset of each light pulse it would temporarily reduce retinal luminance by only about 0.3 log units, followed by partial recovery when the retina has adapted to the new illuminance. Thus, the magnitude of such effects would be small compared to the amplitudes of the flicker light itself. Furthermore, Ashby et al. have studied the impact of changes in pupil size on the protective effect against myopia by using artificial pupils and found no effects [13].

### Potential role of changes in corneal radius of curvature

A limitation in the current study was that corneal radius of curvature was not measured even though it is known that exposing chicks to continuous light can flatten their cornea severely [57]. On the other hand, no relative changes in corneal radius of curvature were found by Ashby et al. [13] when animals were reared under 50, 500 or 15 000 lux. Also Backhouse et al. [29] did not find relative changes in chickens kept at 2,000 lux for 10 hours or at 10,000 lux for 2 hours. Interestingly, Cohen et al. [12] found that corneal radius of curvature responded differently under continuous light and under normal diurnal cycles. Under continuous light, the brighter the lighting is, the flatter the cornea became. But under normal diurnal cycles, the opposite change was observed. All effects were rather small, less than 2D between 10,000 lux and 500 lux for a period of 30 days. In the current study, potential changes in corneal radius of curvature had only a minor effect since changes in refractive state could be explained to 70% to 80% by the changes in vitreous chamber depth (Figure 2).

## Summary

Temporal properties of bright light exposure modulate the impact on deprivation myopia in chickens. For continuous bright light, no significant inhibition occurs below two hours of exposure while the inhibitory effects level off between five and ten hours. With the same total light dose, intermittent bright light provides a stronger effect than continuous light. Deprivation myopia in chickens is completely inhibited by 1∶1 minute square wave light cycles (0.007 Hz), presented in total for five hours a day. However, it should be pointed out that these quantitative data were found in chickens. Although the previous finding that bright light inhibits experimental myopia is an across-species' phenomenon and therefore might be applicable to humans, the exact protocolprobably might not be directly translated into human values. Thus, further amendments are required in clinical studies.

## Supporting Information

Table S1 **Summary of refractive error and ocular biometry data of all groups.**
(XLS)
